# Strain-Specific microRNA Reprogramming of Human Dendritic Cells by Probiotic and Commensal *Escherichia coli* Outer Membrane Vesicles

**DOI:** 10.3390/microorganisms14020323

**Published:** 2026-01-30

**Authors:** Karen Rodas-Pazmiño, Betty Pazmiño-Gómez, Luis Cagua-Montaño, Samuel Valle-Asan, Milena Acosta-Farías, Pedro Javier Fajardo-Aguilar, Priscila Romoleroux-Gutiérrez, Alfonso Jiménez-Gurumendy, Steven Andaluz-Guamán, Edgar Rodas-Neira

**Affiliations:** 1 Faculty of Science and Engineering, Universidad Estatal de Milagro, Milagro 091706, Ecuador; krodasp2@unemi.edu.ec (K.R.-P.); lcaguam@unemi.edu.ec (L.C.-M.); svallea@unemi.edu.ec (S.V.-A.); pfajardoa@unemi.edu.ec (P.J.F.-A.); romoleroux2001@gmail.com (P.R.-G.); jimenezgalf@gmail.com (A.J.-G.); sandaluzg@unemi.edu.ec (S.A.-G.); edgarivan6@hotmail.com (E.R.-N.); 2 Faculty of Animal Science, Universidad Técnica Estatal de Quevedo, Quevedo 120301, Ecuador; jacostaf@uteq.edu.ec

**Keywords:** outer membrane vesicles, *Escherichia coli* Nissle 1917, dendritic cells, microRNAs, mucosal immunology

## Abstract

Outer membrane vesicles (OMVs) are tractable, cell-free microbial outputs that can shape innate immune programs. Here, we compared OMVs from the probiotic *Escherichia coli* Nissle 1917 (EcN) and the commensal strain ECOR12 in a paired within-donor model of human monocyte-derived dendritic cells (Mo-DCs) (N = 20). In the core integrated arm, Mo-DCs were exposed to iDC control, EcN OMVs, or ECOR12 OMVs (10 µg/mL, 24 h) and profiled for maturation markers (CD14, CD83, CD209), cytokines (IL-6, TNF-α, IL-10), and a targeted miRNA panel (miR-155-5p, let-7i-3p, miR-146b-5p, miR-29a-5p). Both OMV types promoted maturation (increased CD83 and reduced CD14), but generated distinct cytokine–miRNA configurations, with ECOR12 tending toward an IL-10–high profile and EcN toward higher IL-6/TNF-α tendencies. Multivariate integration separated conditions into reproducible, strain-specific immune fingerprints, supporting the key take-home that probiotic versus commensal *E. coli* OMVs imprint distinguishable coordinated response states in human DCs. In an extended phenotyping arm, ECOR63 OMVs were evaluated by ELISA and flow cytometry only and were not included in miRNA profiling or integrated PCA due to unavailable matched miRNA measurements.

## 1. Introduction

Inflammatory disorders of the gut are a growing biomedical and public-health challenge because they arise from multi-layer host–microbe interactions that remain incompletely resolved. Inflammatory bowel disease (IBD) has been described as a rising global burden with substantial impacts on quality of life, healthcare costs, and long-term complications [1,2]. Beyond IBD, chronic intestinal inflammation and barrier dysfunction are increasingly linked to altered microbial ecology and abnormal immune tone at mucosal surfaces [3,4]. A key barrier to mechanistic clarity is that microbiota effects are often framed as community-level phenomena, whereas the mucosal immune system responds to discrete molecular cues and particulate structures that can be mapped, measured, and experimentally controlled [5,6]. This mismatch limits our ability to explain why some microbial contexts support homeostasis while others precipitate inflammatory cascades, and it slows the design of targeted microbiota-derived interventions [7,8]. The present study addresses this gap by focusing on a defined, isolatable microbial output: outer membrane vesicles (OMVs) to test strain-resolved immune programming under controlled conditions.

**Figure 1:**
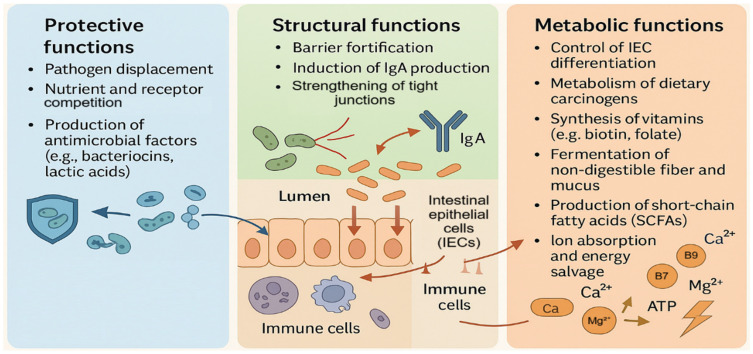
Functional roles of the intestinal microbiota in host homeostasis. Conceptual overview summarizing protective functions (pathogen displacement and antimicrobial factor production), structural functions (barrier fortification, IgA induction, tight-junction strengthening), and metabolic functions (vitamin synthesis, SCFA production, and energy salvage) that collectively shape epithelial–immune equilibrium. Created by the authors; all elements are original.

The intestinal microbiota contributes to host defense, epithelial integrity, and metabolic balance [4,9]. These functions matter for the present work because they converge on barrier maintenance and immune calibration at the epithelial interface. Commensals can displace pathogens, shape mucosal immunity, and reinforce tight junctions, while also producing metabolites and vitamins that influence differentiation and energy homeostasis [3,8]. Intestinal epithelial cells (IECs) coordinate barrier architecture and immune communication through pattern recognition receptors (PRRs), cytokine signaling, and antimicrobial programs [6,10]. Dendritic cells (DCs) integrate luminal information and instruct downstream T-cell polarization toward effector or regulatory programs [11,12]. Thus, epithelial–DC crosstalk is central to homeostasis, and its disruption provides a plausible route to chronic inflammation [6,10] (Figure 1). We use this framework to motivate OMVs as tractable microbial inputs that modulate the epithelial–immune axis.

Microbiota–host signaling does not require whole bacterial cells. Bacterial membrane vesicles mediate microbe–microbe and microbe–host interactions [13]. Gram-negative bacteria release OMVs that traverse mucus and deliver cargos, including LPS, outer membrane proteins, peptidoglycan fragments, nucleic acids, and small RNAs to host cells [14,15]. OMVs are therefore suited for mechanistic testing because they provide quantifiable, standardized packages of microbial information that can be compared across strains [16,17]. Vesicle-mediated signaling is not exclusive to Gram-negative bacteria; Gram-positive bacteria and fungi also produce extracellular vesicles (EVs), reinforcing vesicles as a conserved cross-kingdom communication strategy [18]. In the gut, OMVs provide a tractable platform to link defined microbial cues to host transcriptional and functional outcomes [19,20].

**Figure 2:**
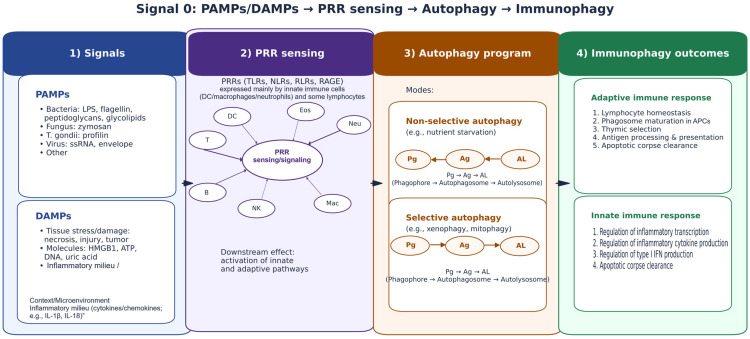
“Signal 0” immune sensing links PAMPs/DAMPs to PRR activation and autophagy-associated immune programs. Diagram integrating microbial and damage signals (PAMPs/DAMPs), recognition by PRRs (e.g., TLRs, NLRs, RLRs, RAGE), downstream autophagy pathways (non-selective vs. selective), and resulting effects on innate and adaptive immune responses (“immunophagy”). Created by the authors; all elements are original.

OMV effects vary by origin, cargo, and responding cell type. Pathogen-derived vesicles can amplify inflammation and contribute to severe phenotypes [21,22], whereas commensal/probiotic-derived OMVs can engage PRRs in ways that support barrier reinforcement and balanced immune activation [23,24]. OMVs can enter IECs via endocytic routes such as clathrin-dependent internalization, producing outcomes ranging from DNA damage responses to immune pathway activation, depending on cargo and cell context [25]. OMV signaling intersects innate recognition (e.g., TLRs and NOD-like receptors), inflammasome pathways, cytokine networks, and epithelial stress programs [5,26]. These pathways connect to autophagy-related processes involved in antigen handling, inflammation, and cellular homeostasis, particularly under “signal 0” sensing driven by PAMPs and DAMPs [27,28] (Figure 2). This framework is retained here to position OMVs as structured PAMP-bearing inputs that can engage PRR-linked cascades shaping downstream immune states.

Among Gram-negative organisms, *Escherichia coli* is particularly informative because strains span a continuum from commensals to opportunists, and some are used clinically as probiotics. *E. coli* Nissle 1917 (EcN) has a long history of therapeutic use and traits supporting gut persistence and host benefit, including colonization factors, microcin production, and immunomodulatory potential [29,30]. Fimbriae contribute to biofilm formation and colonization [31], and capsule-associated features can shape epithelial PRR responses and MAPK-dependent cytokine induction [32]. However, EcN is not uniformly “non-inflammatory”; effects are context- and pathway-dependent, motivating direct measurement of how EcN-derived structures, particularly OMVs, shape immune programs rather than assuming a fixed probiotic phenotype [29,33]. Phylogenetic frameworks and reference collections further support strain-resolved interpretation of how commensal background can influence vesicle composition and host responses [34,35]. This strain-spectrum logic motivates our comparison of EcN OMVs with commensal *E. coli* OMVs.

Proteomic and functional studies support the feasibility and relevance of OMV-based mechanistic analysis. Proteomic profiling indicates complex OMV compositions consistent with selective cargo loading [36,37]. In vivo and ex vivo work shows that OMVs from commensal and probiotic *E. coli* can activate immune and defense programs in the intestinal mucosa, including pathways linked to innate sensing and barrier regulation [38]. In experimental colitis models, EcN OMVs have been associated with anti-inflammatory effects [33], while other work demonstrates that OMVs can elicit strong inflammatory outputs depending on cargo and immune compartment [17,21]. This mixed landscape motivates the core question of this study: whether OMVs from probiotic versus commensal *E. coli* produce distinct, reproducible immune response configurations in human antigen-presenting cells under controlled exposure conditions.

DCs are a central node for resolving such strain-specific programming. DCs integrate PRR signals, inflammasome activity, and cytokine cues to guide T-cell differentiation, B-cell help, and tolerance induction [11,12]. In addition to surface markers and cytokines, DC states are shaped by microRNAs (miRNAs), which regulate the magnitude and duration of inflammatory signaling [39,40]. miR-155 is induced during inflammatory activation and modulates cytokine production [41], including IL-1 pathway regulation in activated monocyte-derived DCs [42]. Conversely, IL-10-dependent miR-146b can suppress TLR4 signaling as a negative-feedback mechanism [43]. Because OMVs deliver strain-specific combinations of PAMPs and can differentially engage PRR/TLR signaling (notably TLR4/TLR2), we posited that downstream NF-κB/AP-1 outputs would be tuned by miRNA feedback circuits calibrating innate immune setpoints [44,45,46,47,48]. We therefore advanced a directional working hypothesis aligned with our readouts: OMVs that drive a more pro-inflammatory output (higher IL-6/TNF-α) would preferentially increase activation-linked miRNAs (miR-155 and let-7i), whereas OMVs associated with a more regulatory profile (higher IL-10) would preferentially engage regulatory miRNAs (miR-146b and miR-29a), linking early cytokine release to post-transcriptional programming [44,45,46,47,48].

miRNAs also influence epithelial barrier outcomes that are central to gut disease biology. miR-29a is associated with increased intestinal permeability and tight-junction regulation in clinical and experimental contexts [49,50]. Inhibition of miR-29a restores barrier-associated proteins (e.g., ZO-1 and claudins) in diarrhea-predominant models [51]. let-7 family members regulate TLR4 expression and influence epithelial immune responses to infection-related stimuli [52]. Together, these findings support the mechanistic premise that OMVs can reshape mucosal-relevant outcomes via PRR-triggered cytokines [5] and miRNA networks that determine intensity and persistence of immune activation [39,40,53]. This premise underlies our focus on combined cytokine and miRNA responses as an integrated DC programming output.

Consistent with this rationale, emerging evidence shows that microbiota-derived vesicles can imprint miRNA-linked immune states. Transcriptomic profiling indicates that DCs respond to gut microbiota vesicles with distinct miRNA signatures [54]. Vesicles from EcN and gut-resident *E. coli* strains can differentially modulate human DCs and influence downstream T-cell responses, suggesting strain specificity at the level of adaptive instruction [55]. Reviews consolidate microbiota EVs as relevant mediators of gut homeostasis and disease, emphasizing immunoregulatory signaling, barrier effects, and cargo-driven mechanisms [20,56,57]. However, a recurrent limitation is that vesicle studies often emphasize single mediators, while OMV responses are multivariate and likely coordinated across inflammatory and regulatory axes [58,59]. This motivates the analytical strategy used here: identifying response “fingerprints” rather than interpreting isolated markers.

Dimensionality reduction and clustering are well suited to extract such fingerprints from correlated immune variables. PCA summarizes correlated cytokine/miRNA variation into interpretable axes, and biplots identify which variables structure condition separation [60,61,62]. Hierarchical clustering and heatmaps reveal coherent response modules, and annotation-rich heatmap frameworks facilitate biological interpretation [63,64,65]. For multivariate outcomes, MANOVA provides formal tests of condition effects, with classical robustness considerations and modern alternatives when assumptions are challenged [66,67]. Finally, transparent effect-size reporting is essential in biological systems where statistical significance may not correspond to biological relevance, motivating careful interpretation of effect-size metrics across model structures [68,69]. These tools are applied here to define strain-resolved cytokine–miRNA configurations in a donor-paired DC model.

**Figure 3:**
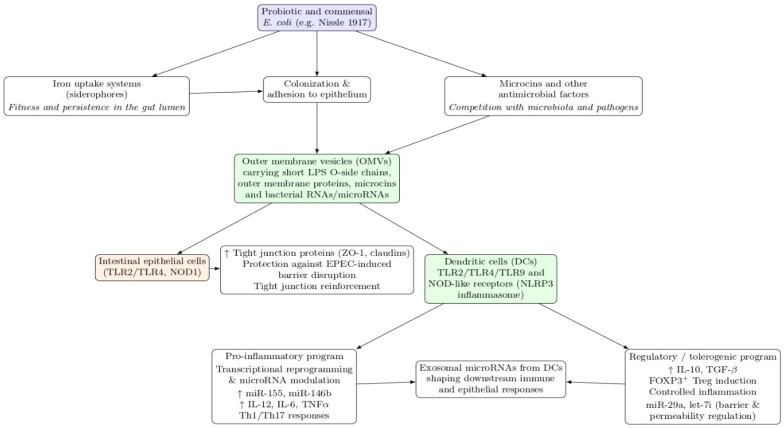
Working model of probiotic/commensal *E. coli* traits converging on OMV-mediated host modulation. Schematic connecting strain attributes (iron uptake, adhesion/colonization, microcins) to OMV cargo delivery (LPS, outer membrane proteins, RNAs/small RNAs) and downstream outcomes in intestinal epithelial cells and dendritic cells, including tight-junction reinforcement and polarization toward inflammatory or regulatory programs shaped by cytokines and miRNA modulation.

Within this framework, EcN OMVs are particularly compelling because they package probiotic traits, colonization-associated factors, microcins, and immunologically active surface components into a stable, cell-free format deliverable to epithelial and immune compartments without live bacterial replication [29,36]. Commensal *E. coli* strains provide the required counterpoint to distinguish probiotic-associated patterns from broader commensal signaling [34,35]. Prior work indicates that OMVs from probiotic and commensal *E. coli* activate innate pathways such as NOD1-mediated responses in epithelial cells [23], and EcN vesicles can protect against epithelial barrier dysfunction induced by enteropathogenic *E. coli* [70,71]. Evidence that probiotic-derived OMVs influence macrophage polarization and antimicrobial activity further supports vesicles as active immunological agents with compartment-specific outputs [72,73]. These observations support a study-specific working model linking strain attributes to OMV cargo and to downstream epithelial and DC programs shaped by cytokines and miRNA regulation [16,17,74] (Figure 3).

Accordingly, we contrasted OMVs from the probiotic EcN with OMVs from commensal *E. coli* in a two-tier design. ECOR12 served as the prespecified commensal comparator for integrated cytokine–miRNA analyses, while ECOR63 was included as an additional commensal reference to capture commensal heterogeneity in cytokine secretion and maturation marker phenotypes. This structure enables strain-resolved immune fingerprints to be defined in the matched multi-omic dataset (EcN vs. ECOR12 vs. control) while contextualizing commensal diversity (ECOR63) in the phenotyping assays.

**Figure 4:**
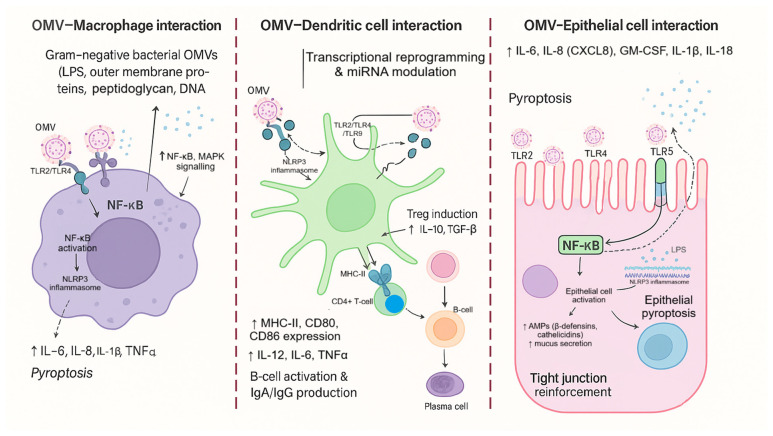
Cell-type-resolved pathways of OMV interaction at the intestinal interface. Summary of OMV-driven signaling in macrophages (NF-κB/MAPK activation and inflammasome-linked inflammation), dendritic cells (transcriptional/miRNA reprogramming, antigen presentation, and tolerance vs. effector skewing), and epithelial cells (PRR signaling, antimicrobial programs, mucus, tight-junction reinforcement, and pyroptosis-related outcomes), highlighting how vesicle effects depend on the responding cell compartment.

In this study, we examine how OMVs derived from EcN and a defined commensal comparator (ECOR12) shape mucosal-relevant immune signaling with an explicit focus on DC reprogramming and miRNA-linked regulation. To prevent ambiguity in strain framing, ECOR12 was treated as the prespecified commensal comparator for the integrated core arm (iDC control, EcN OMVs, ECOR12 OMVs), which included cytokines, flow cytometry, miRNA RT-qPCR, and cytokine–miRNA multivariate integration. ECOR63 was included as an additional commensal reference in an extended phenotyping arm (iDC control, EcN, ECOR12, ECOR63) to capture commensal heterogeneity in cytokine secretion and maturation marker phenotypes, while miRNA profiling and cytokine–miRNA integrated multivariate models remained restricted to the core arm due to unavailable matched miRNA measurements for ECOR63. We leverage current understanding of innate recognition pathways [5], IEC–DC mucosal crosstalk [10,11], and the emerging role of microbiota EVs in gut homeostasis [56,57] to frame OMVs as modular immune inputs capable of inducing coordinated cytokine–miRNA response states. The schematic figures are retained in the same order to support the experimental logic: microbiota functions relevant to barrier integrity and immune tone (Figure 1); the PAMP/DAMP–PRR axis (Figure 2); the strain attribute–to–OMV cargo framework (Figure 3); and compartment-specific OMV interaction pathways across macrophages, DCs, and epithelium (Figure 4).

Therefore, the objectives of this study were threefold. First, we characterized how OMVs from a probiotic strain (EcN) and two commensal strains (ECOR12 and ECOR63) shape Mo-DC maturation and cytokine production in a paired, donor-blocked design. Second, we profiled a targeted panel of immune-related miRNAs for the core integrated arm (iDC control, EcN OMVs, ECOR12 OMVs) and integrated miRNA and cytokine readouts using multivariate analysis to define strain-specific “fingerprints.” Third, we used the extended phenotyping arm (adding ECOR63 OMVs for ELISA/flow cytometry only) to contextualize the cytokine patterns with protein-level readouts of TGF-β1 and DC surface markers.

## 2. Materials and Methods

### 2.1. Experimental Design

The study comprised a core integrated arm comparing iDC control, EcN OMVs, and ECOR12 OMVs (used for cytokines, flow cytometry, miRNA RT-qPCR, and integrated multivariate analyses; N = 20 donors; N = 60 donor–condition observations). In addition, an extended phenotyping arm included ECOR63 OMVs alongside iDC control, EcN, and ECOR12 for cytokine ELISA and flow cytometry (and OMV protein profiling) [14,15,16,17], enabling a four-condition comparison (N = 20 donors; N = 80 donor–condition observations). miRNA profiling and cytokine–miRNA integrated multivariate models were restricted to the core arm due to the availability of matched miRNA measurements (Table 1).

### 2.2. Evaluated Variables

Outcome selection was guided by (i) the established role of dendritic cells as integrators of microbial sensing and T-cell instruction [10,11,12], and (ii) evidence that microbiota and *E. coli*–derived vesicles can reprogram DC cytokines and microRNA (miRNA) networks [54,55]. Cytokines (IL-6, IL-10, TNF-α) were quantified to capture canonical maturation-associated outputs, while flow cytometry markers (CD14, CD83, CD209) were used to phenotype the iDC→mDC transition [5,27]. miRNA targets were quantified by RT-qPCR to reflect key regulatory layers of innate signaling [41,42,43,44,45,47,48]. The complete panel of evaluated variables and their measurement modality is listed in Table 2.

### 2.3. Biological Material and Dendritic-Cell Generation

Peripheral blood from healthy donors was used as the source of peripheral blood mononuclear cells (PBMCs). PBMCs were isolated by density gradient separation using Histopaque^®^ 1077 (Sigma-Aldrich, St. Louis, MO, USA), followed by washing and cell counting. CD14^+^ monocytes were isolated by positive selection (MidiMACS system; LS columns; CD14 Microbeads), then differentiated into immature monocyte-derived dendritic cells (Mo-DCs; iDCs) in Mo-DC differentiation medium supplemented with recombinant human IL-4 and GM-CSF under standard incubator conditions (37 °C, 5% CO_2_) for approximately 6–7 days. This differentiation strategy aligns with established approaches for generating monocyte-derived DCs for microbial/vesicle immunomodulation studies [21,27] and provides a biologically relevant platform to assess OMV-driven DC programming [24,55]. Mo-DCs were generated from N = 20 independent healthy donors (biological replicates), where each donor constituted one independent Mo-DC differentiation. For each donor, iDCs were split across conditions in a paired (within-donor) design to control inter-individual variability. For the core integrated arm (Control, EcN OMVs, and ECOR12 OMVs), this paired design yielded N = 60 donor–condition observations (20 × 3) for miRNA profiling and multivariate integration; ECOR63 OMVs were included as an extended phenotyping arm using the same donor-derived Mo-DC preparations (N = 20 donors) for OMV characterization, cytokine ELISA, and flow cytometry, but were not included in miRNA analyses or integrated PCA because matched miRNA measurements were not available for ECOR63.

The study was conducted in accordance with the Declaration of Helsinki and approved by the Bioethics 631 Committee of the University of Barcelona (Institutional Review Board: IRB00003099; approval year: 2020). Written informed consent was obtained from all participants prior to blood collection. Healthy donors (N = 20) were enrolled according to the following inclusion criteria: adults aged 18–55 years; clinically healthy at the time of donation; no self-reported acute infectious symptoms within the previous 14 days; and ability to provide written informed consent. Exclusion criteria included: history of chronic inflammatory or autoimmune disease, immunodeficiency, current pregnancy or breastfeeding, use of systemic immunomodulatory therapy (e.g., corticosteroids or biologics) within the preceding 30 days, antibiotic use within the preceding 30 days, and vaccination within the preceding 14 days. Donor samples were anonymized prior to processing.

### 2.4. OMV Production, Isolation, Sterility Control, and Pre-Use Characterization

OMVs were produced from *E. coli* strains grown on LB agar and incubated at 37 °C. After growth, bacteria were removed by centrifugation (10,000× *g*, 30 min, 4 °C) and the supernatant was sterile-filtered (0.2 µm) while kept at 4 °C (on ice). The filtrate was concentrated using Centricon^®^ Plus-70 devices (10 kDa cutoff; swinging bucket rotor at 3500× *g*, 20 min, 4 °C; MilliporeSigma, Burlington, MA, USA) to reduce ~1 L to ~8 mL. Residual bacteria were removed by 0.2 µm syringe filtration, and OMVs were pelleted by ultracentrifugation (RCF = 242,000× *g*, 1 h, 4 °C; Beckman Coulter Optima L-90K ultracentrifuge (Beckman Coulter, Brea, CA, USA), Type 45 Ti fixed-angle rotor; equivalent to 45,000 rpm). Pellets were resuspended in sterile PBS, sterility was verified by plating on LB agar (37 °C, 48 h), OMV protein was quantified by the Lowry method, and aliquots were stored at −20 °C until use. This workflow is consistent with the principle that OMVs represent a structured output of Gram-negative bacteria, with bioactivity that depends on clean isolation and standardized input dosing [15,36,37,73]. OMV inputs were normalized by total protein; particle size/concentration (e.g., NTA/DLS) and endotoxin (LPS) quantification were not performed, and this is now stated explicitly for transparency.

For structural and compositional quality control prior to stimulation assays, OMVs were processed for Cryo-TEM in a dedicated microscopy facility using cryogenic conditions (−170 to −175 °C) and electron acceleration voltage (200 kV), following the study’s microscopy preparation pipeline [22]. In addition, OMV protein profiles were assessed by SDS-PAGE (10% acrylamide; ~10 µg protein loaded; run at 20 mA; Coomassie staining) based on prior laboratory protocols used for OMV protein profiling [25].

### 2.5. Dendritic-Cell Stimulation with OMVs

After Mo-DC differentiation, iDCs were washed with PBS and resuspended in Mo-DC medium. Cells were then stimulated with OMVs derived from intestinal *E. coli* strains at 10 µg/mL for 24 h (37 °C; 5% CO_2_). This exposure paradigm was chosen to mimic physiologically relevant vesicle–immune cell contact at mucosal interfaces, where OMVs can deliver immunostimulatory and immunoregulatory cargos [5,16,20,57,74].

### 2.6. Data Acquisition Equipment and Measurement Settings

To ensure transparency in data acquisition, the main instruments and key settings are summarized in Table 3.

### 2.7. Flow Cytometry Phenotyping of Dendritic-Cell Maturation

DC maturation status was evaluated by flow cytometry using fluorescent monoclonal antibodies targeting CD14 (monocyte marker), CD83 (maturation marker), and CD209/DC-SIGN (immature DC-associated marker). Data were acquired in a dedicated cytometry facility, and iDCs served as the phenotypic baseline control. Marker selection is consistent with established immunophenotyping approaches used to separate immature and mature Mo-DC states [27], and supports mechanistic interpretation of OMV-driven maturation alongside cytokine/miRNA readouts [11,55].

Gating and QC. Events were first gated on FSC/SSC to select the main cell population, followed by doublet exclusion using FSC-A versus FSC-H (singlets). No viability dye was used; thus, viability exclusion was not applied. Compensation was performed using single-stained controls, and gates were defined using unstained and/or fluorescence-minus-one controls as appropriate. Flow-cytometry results are presented as representative histogram; no MFI or % positive summary metrics were used for inferential statistics in this study.

### 2.8. Cytokine Quantification by ELISA

Culture supernatants were collected after 24 h stimulation and assessed for IL-6, IL-10, and TNF-α secretion using BD Biosciences ELISA kits (BD Biosciences, San Jose, CA, USA), following manufacturer protocols and previously established laboratory procedures. Cytokine selection reflects the central role of these mediators in DC maturation and inflammatory/regulatory polarization downstream of pattern recognition receptor activation [5,6]. Each ELISA measurement was run in technical triplicate per donor–condition, and triplicates were averaged to obtain one value per donor–condition prior to statistical inference.

### 2.9. miRNA Extraction and RT-qPCR

Total RNA enriched for small RNAs was extracted from dendritic cells after OMV exposure using the miRNeasy^®^ Mini Kit (QIAGEN, Hilden, Germany). RNA quantity and purity were assessed by NanoDrop^®^ ND-1000 spectrophotometry (Thermo Fisher Scientific, Wilmington, DE, USA) (A260/A280). Samples were stored at −80 °C until analysis. Reverse transcription was performed using miRCURY^®^ LNA^®^ RT Kit (QIAGEN, Hilden, Germany), and qPCR was conducted using miRCURY LNA miRNA PCR assays (QIAGEN, Hilden, Germany). The miRNA primers used in this study are provided in Appendix A (Table A1). UNISP was included as a spike-in control to monitor extraction/RT efficiency. Relative expression was normalized to the endogenous reference U6 snRNA and computed using the 2^−ΔΔCt^ method. This approach is widely used for miRNA quantification in immune-cell activation contexts, where miRNAs are established regulators of TLR/IL-1 signaling and inflammatory feedback control [41,42,43,44,45,47,48]. RT-qPCR reactions were performed in technical triplicate per donor–condition. Ct values were quality-checked and averaged across technical replicates prior to ΔCt/ΔΔCt computation and log2 transformation for downstream statistical testing.

### 2.10. Data Analysis

Statistical analysis and unit of inference. Biological replication corresponded to independent donors (N = 20), with each donor providing one independent Mo-DC differentiation. All experimental conditions were evaluated in a paired within-donor design, such that the donor constituted the statistical subject/block. Where technical triplicates were performed (ELISA and RT-qPCR) [54,58,59], replicate wells were averaged to a single value per donor–condition prior to inferential statistics to avoid pseudoreplication; thus, the unit of inference was the donor–condition observation. Software and reproducibility. Analyses were conducted in R (v4.5.0; R Foundation for Statistical Computing, Vienna, Austria) using RStudio IDE (2025.09.2) using standard functions for repeated-measures ANOVA/mixed models, MANOVA, PCA (prcomp), clustering (hclust/kmeans), and plotting. Missing values were handled by complete-case analysis within each model, and outliers were inspected (boxplots/standardized residuals) but were not removed unless attributable to documented technical failure.

For the core integrated arm (iDC control, EcN, ECOR12; N = 60), univariate outcomes were analyzed using repeated measures ANOVA (within-subject factor: condition; subject: donor) or, equivalently, a linear mixed effects model with donor as a random intercept. Post hoc pairwise contrasts were performed as donor-matched comparisons with multiplicity control (Holm). Cytokine–miRNA multivariate inference was performed on the core dataset using donor blocked MANOVA (Pillai’s trace), with PCA and hierarchical clustering used for exploratory visualization (Table 4).

For the extended phenotyping arm (iDC control, EcN, ECOR12, ECOR63; N = 80), cytokines and flow-cytometry marker summaries were analyzed with the same repeated-measures/mixed-effects framework (within-donor factor: condition; donor as subject/random effect), followed by donor-matched post hoc contrasts.

### 2.11. Null and Working Hypotheses (Pre-Specified)

Hypotheses were defined at both multivariate and variable-specific levels, consistent with the concept that OMV exposure can shift coordinated immune programs rather than a single endpoint [15,57,74].

Null hypothesis (H0, multivariate): The joint response vector (cytokines + miRNA expression profile ± phenotypic markers) does not differ among groups (control iDC vs. EcN OMVs vs. ECOR12 OMVs). Operationally, H0 is evaluated by MANOVA and rejected when *p* < 0.05 using Pillai’s trace, given its documented robustness [66], with robust alternatives considered under assumption violations [67].

Working/alternative hypothesis (H1): At least one OMV condition produces a statistically detectable shift in the multivariate immune response profile relative to control, and/or EcN and ECOR12 OMVs elicit distinguishable response fingerprints. This expectation is biologically grounded in the strain-specific immunomodulatory capacity of *E. coli* OMVs and microbiota-derived vesicles [24,25,54,55] and in miRNA-mediated tuning of innate signaling circuits [42,43,45].

All a priori hypotheses and inferential statistics for cytokine–miRNA integration were applied to the core integrated dataset (iDC control, EcN, ECOR12). ECOR63 was included as an extended phenotyping condition for cytokine ELISA and flow cytometry (and OMV protein profiling), but it was not included in miRNA profiling or cytokine–miRNA integrated multivariate models due to unavailable matched miRNA measurements.

## 3. Results

### 3.1. Characterization of OMVs: Cryo-TEM and SDS-PAGE

Before assessing OMV biological effects, OMV preparations were characterized. Isolated OMVs were quantified using the Lowry protein assay [73], and the resulting preparations were subsequently processed for Cryo-TEM visualization.

Protein concentrations (Lowry assay) and the corresponding volume required to load 10 µg OMV protein per lane are summarized in Table 5 (final lane volume 20 µL with 4× loading buffer).

**Figure 5:**
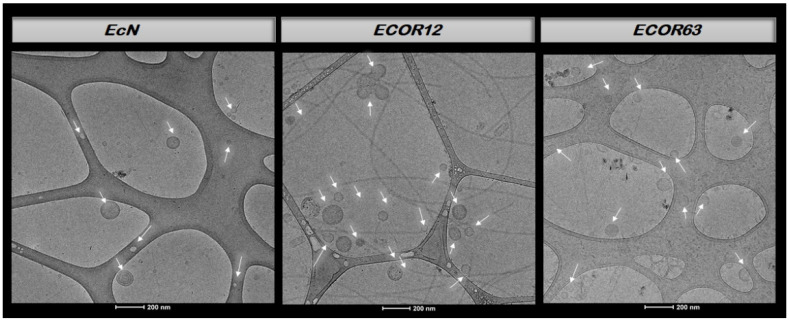
Cryo-TEM images of OMVs isolated from different strains. EcN OMVs, ECOR12 OMVs, ECOR63 OMVs. Vesicles are indicated by arrows.

Representative Cryo-TEM micrographs of OMVs from each strain are presented in Figure 5. Cryo-TEM imaging was performed on 2 independent OMV preparations per strain, with 10 fields/grid examined; vesicle morphology and the observed size range were consistent across preparations.

As shown in Figure 5, vesicles obtained from the probiotic strain EcN and from the two commensal strains (ECOR12 and ECOR63) exhibited a round morphology with a clearly defined membrane, and in some cases, double-membrane structures were observed. OMVs typically ranged from ~20–200 nm [21].

#### Protein Profile of OMVs (SDS-PAGE)

In addition to Cryo-TEM, the OMV protein profile of each sample was analyzed to relate banding patterns to the phylogenetic background of commensal vs. probiotic strains. A BenchMark™ Pre-Stained Protein Standard (Cat. No. 10747-012; Invitrogen, Carlsbad, CA, USA) was used as the molecular weight marker.

**Figure 6:**
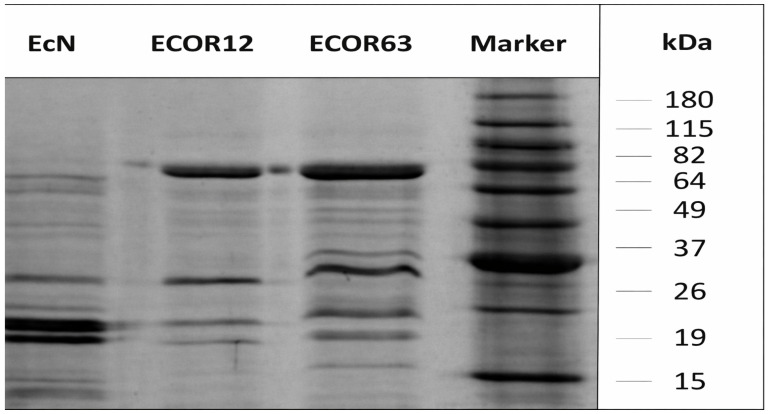
Electrophoretic separation of proteins present in OMVs. OMVs were separated on a 10% acrylamide gel and visualized by Coomassie Blue staining (~10 µg OMV protein loaded per lane; final lane volume 20 µL). The molecular weight marker “Marker” indicates molecular weights labeled on the side.

The SDS-PAGE separation of OMV-associated proteins is shown in Figure 6.

The gel banding patterns differed among EcN, ECOR12, and ECOR63. EcN displayed a profile distinct from the commensal strains and showed a set of lower molecular weight bands (group A) relative to the other two strains. ECOR12 showed a pattern distinct from EcN and ECOR63, consistent with strain-dependent differences in OMV protein cargo; however, banding patterns alone cannot be used to infer phylogenetic background.

### 3.2. Maturation Profile of Dendritic Cells Stimulated with OMVs from Commensal and Probiotic Strains

DC maturation was evaluated using two complementary approaches: (i) ELISA quantification of secreted cytokines and (ii) flow cytometry assessment of surface maturation markers. Because ECOR63 was included as an additional commensal reference, cytokine ELISA and flow cytometry comparisons include EcN, ECOR12, and ECOR63; however, miRNA profiling and integrated multivariate analyses were restricted to the prespecified paired core design (iDC control, EcN OMVs, ECOR12 OMVs) due to the availability of matched miRNA measurements.

#### 3.2.1. Cytokine Analysis by ELISA

**Figure 7:**
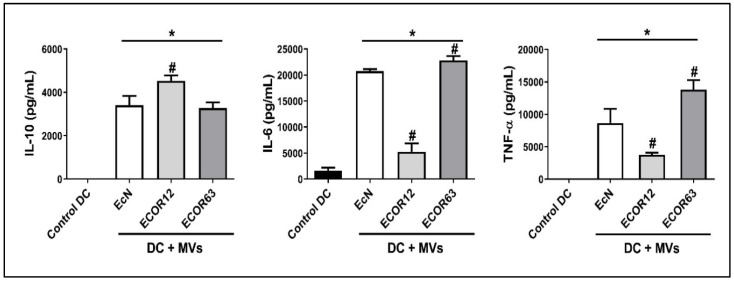
Cytokine secretion in the extended phenotyping arm. ELISA quantification of IL-6, IL-10, and TNF-α (pg/mL) in culture supernatants after 24 h stimulation with 10 µg/mL OMVs. Data are shown as mean ± SD. Core paired dataset: iDC control, EcN, ECOR12 (N = 20 donors). Exploratory arm: ECOR63 (N = 20 donors; phenotyping arm dataset, N = 80 donor–condition observations). Statistical testing was performed using repeated-measures ANOVA (donor as subject; within-subject factor: condition), followed by paired post hoc contrasts with Holm adjustment (α = 0.05). Symbols indicate: * *p* < 0.05 vs. iDC control; # *p* < 0.05 vs. EcN.

To assess maturation-associated cytokine release after stimulation with OMVs from different strains, cytokines were measured by ELISA, focusing on IL-6, IL-10, and TNF-α. The maturation profile of immature DCs (iDC; control) was assessed by ELISA. In the extended phenotyping arm (iDC control, EcN, ECOR12, ECOR63), iDC controls were compared against DCs exposed to OMVs from EcN, ECOR12, and ECOR63. The ELISA quantifications are shown in Figure 7.

As shown in Figure 7, iDC controls exhibited very low cytokine release compared with OMV-stimulated DCs. For IL-10, ECOR12 OMVs induced a significantly higher release relative to EcN and ECOR63 OMVs. For IL-6, ECOR12 OMVs induced lower release than EcN and ECOR63, with ECOR63 showing the highest IL-6. For TNF-α, ECOR12 OMVs induced lower release than EcN and ECOR63, with ECOR63 again showing the strongest significance.

#### 3.2.2. DC Maturation by Flow Cytometry

**Figure 8:**
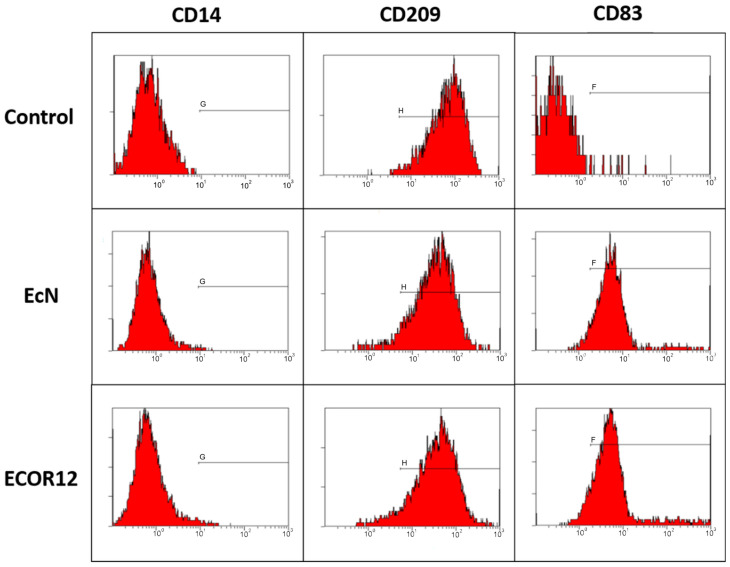
Representative flow-cytometry histograms of CD14, CD209, and CD83 in Mo-DCs under iDC control and after stimulation with OMVs from EcN, ECOR12, or ECOR63 (10 μg/mL, 24 h). Histograms are shown as representative donor profiles to illustrate maturation-associated shifts in marker fluorescence.

Immature monocyte-derived DCs were stimulated with 10 μg/mL OMVs from EcN, ECOR12, or ECOR63 for 24 h, alongside iDC controls, and stained for CD14, CD209, and CD83 (anti-CD14-FITC, anti-CD209-APC, anti-CD83-PE). Representative histograms are shown in Figure 8.

As indicated in Figure 8, the maturation marker CD83 exhibited higher fluorescence in OMV-treated conditions relative to the control, supporting successful DC maturation after OMV exposure.

### 3.3. Identification of miRNA Expression in DCs Stimulated with OMVs from Commensal and Probiotic Strains

**Figure 9:**
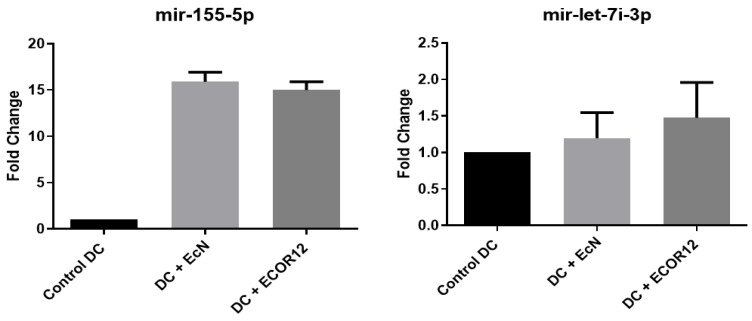
miR-155-5p and let-7i-3p expression in Mo-DCs stimulated with EcN and ECOR12 OMVs (core arm). The data represent N = 20 donors in a paired design. RT-qPCR was performed in technical triplicate per donor–condition and averaged prior to ΔΔCt/log2 analysis. Statistics: repeated-measures ANOVA with Holm-adjusted paired contrasts.

DCs were stimulated with OMVs from EcN and ECOR12 to extract RNA and evaluate the expression of selected miRNAs. The expression results for miR-155-5p and let-7i-3p are shown in Figure 9.

**Figure 10:**
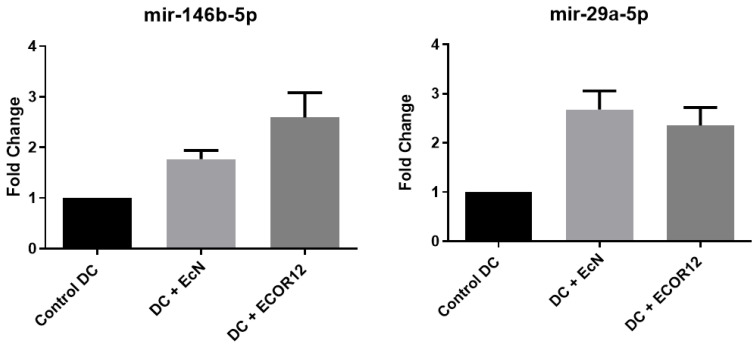
miR-146b-5p and miR-29a-5p expression in Mo-DCs stimulated with EcN and ECOR12 OMVs (core arm). The data represent N = 20 donors in a paired design. RT-qPCR technical triplicates were averaged per donor–condition prior to inference. Statistics: repeated-measures ANOVA with Holm-adjusted paired contrasts.

For miR-155-5p and let-7i-3p, OMVs from both EcN and ECOR12 induced significant expression relative to unstimulated controls. No statistically significant differences were detected between strains (EcN vs. ECOR12) for miR-155-5p; for let-7i-3p, ECOR12 showed a higher trend without reaching significance. Differential expression results for miR-146b-5p and miR-29a-5p are shown in Figure 10.

### 3.4. PCA Biplot with k-Means Clustering (k = 3)

**Figure 11:**
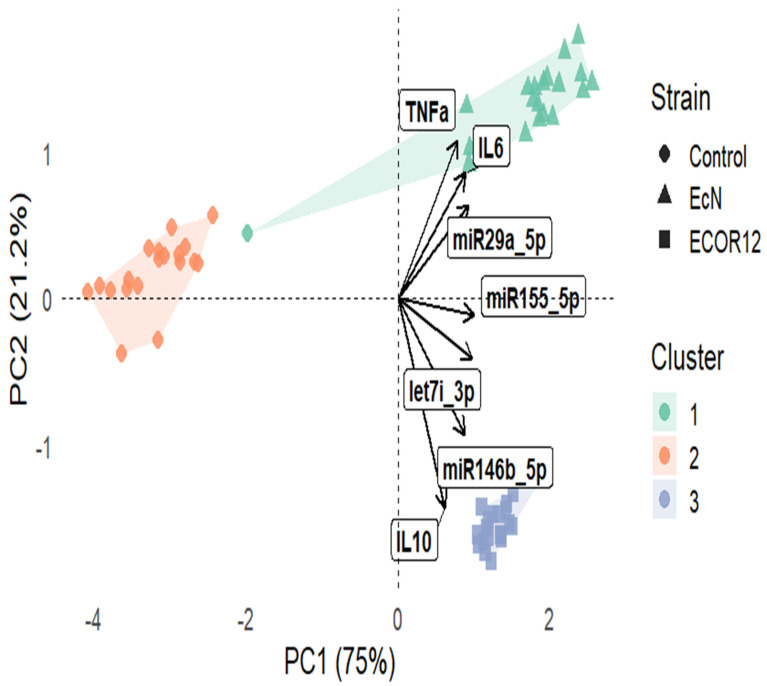
PCA biplot with k-means clustering (k = 3). PCA of cytokines (IL-6, IL-10, TNF-α) and miRNAs (log2 miR-155-5p, let-7i-3p, miR-146b-5p, miR-29a-5p). Points correspond to donor–condition observations (N = 60 = 20 donors × 3 core conditions), computed after averaging technical triplicates per donor–condition, shapes indicate core arm (Control, EcN, ECOR12), and colors represent k-means clusters (k = 3). Arrows correspond to variable loadings (vectors). Axis labels report explained variance (PC1 = 75.0%, PC2 = 21.2%).

A principal component analysis (PCA) was applied to the combined dataset of cytokines (IL-6, IL-10, TNF-α; pg/mL) and miRNA expression (log2 miR-155-5p, let-7i-3p, miR-146b-5p, miR-29a-5p). The first two components captured most of the total variance, with PC1 = 75.0% and PC2 = 21.2% (cumulative 96.2%), indicating a strong low-dimensional structure suitable for visualization. In the PCA biplot (Figure 11), samples separated clearly across the PC1–PC2 space, and k-means (k = 3) produced three clusters that were largely concordant with strain level structure. Vector loadings showed that IL-6 and TNF-α aligned with the upper right region of the ordination (positive contribution in the direction of higher inflammatory response), whereas IL-10 loaded toward the lower portion of the plot, consistent with a distinct axis of variation driven by anti-inflammatory signaling. miRNA vectors (notably miR-29a-5p, miR-155-5p, miR-146b-5p, let-7i-3p) projected strongly away from the origin, supporting their high discriminatory contribution to sample separation.

### 3.5. Hierarchical Clustering Heatmap (Integrated Cytokines + miRNAs)

**Figure 12:**
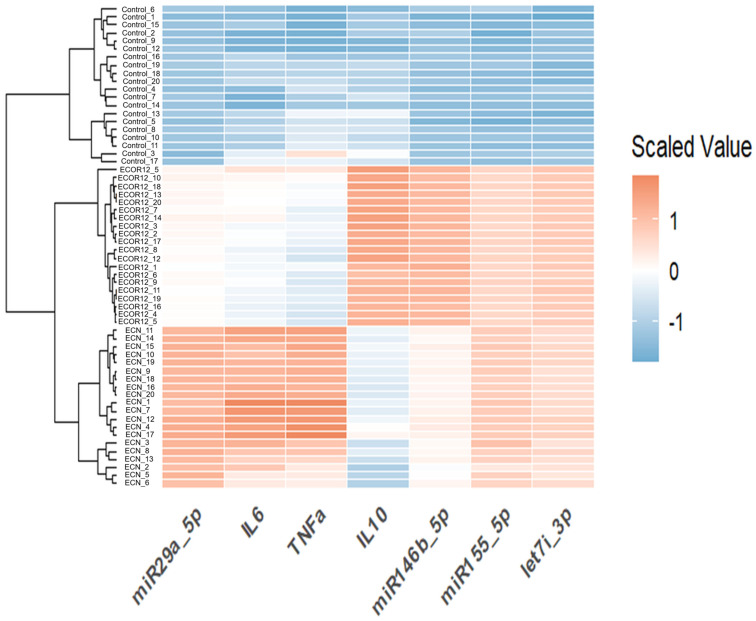
Hierarchical clustering heatmap (strain-annotated). Heatmap of row-scaled biomarker levels (z-scores) for cytokines (IL-6, IL-10, TNF-α) and miRNAs (log2 miR-155-5p, let-7i-3p, miR-146b-5p, miR-29a-5p). Rows are samples (strain_rep), columns are variables. Both rows and columns were hierarchically clustered using complete linkage. The left annotation bar indicates strain (Control, EcN, ECOR12). Color scale denotes scaled value (blue = lower than mean; red = higher than mean).

To evaluate global patterning and similarity structure across samples, we generated a heatmap using row-scaled (z-scored) values and performed hierarchical clustering (complete linkage) for both samples and variables. The resulting heatmap (Figure 12) revealed three dominant blocks aligned with the strain annotation bar, showing consistent within-strain profiles and strong between-strain contrasts. Controls exhibited broadly lower scaled values across most biomarkers (predominantly blue), whereas EcN and ECOR12 displayed elevated signatures across multiple variables (predominantly red), with clear differences in which markers were most enhanced. Variable clustering further indicated that biomarkers co-varied in biologically interpretable modules (e.g., inflammatory cytokines vs. regulatory miRNA patterns), supporting the PCA separation and motivating a formal multivariate test.

### 3.6. MANOVA (Global Effect of Strain) and Univariate Follow-Up with Effect Sizes (ηp^2^)

Given the coordinated behavior among biomarkers (as evidenced by PCA and hierarchical clustering), we tested whether strain produced a significant multivariate shift in the combined cytokine + miRNA response using a donor-blocked multivariate linear model (biomarkers ~ strain + donor). The multivariate model was highly significant using Pillai’s trace: Pillai = 1.9925, Approx. F(14,66) = 1244.85, *p* = 3.42 × 10^−74^ (Table 6).

To interpret the multivariate effect, we performed repeated-measures ANOVAs for each biomarker (donor as subject/block; within-subject factor: condition). All biomarkers showed strong strain effects (Table 7). Effect sizes were large across cytokines (ηp^2^ = 0.845–0.941) and especially pronounced for miRNAs (ηp^2^ = 0.990–0.996), indicating that miRNA expression contributed the strongest discriminatory power between conditions.

## 4. Discussion

Inflammatory disorders of the gastrointestinal tract continue to rise in prevalence and societal impact, and they remain difficult to treat precisely because disease trajectories emerge from multilayered interactions among microbial communities, epithelial barrier physiology, and innate–adaptive immune crosstalk [1,2,3,4]. In this context, the present study is relevant because it focuses on a mechanistically “addressable” unit of microbiota–host communication: bacterial extracellular vesicles, specifically Gram-negative outer membrane vesicles (OMVs). OMVs can package and deliver immunologically active cargo (e.g., lipopolysaccharide, outer membrane proteins, peptidoglycan fragments, DNA/RNA, and small RNAs) to epithelial and immune cells, enabling bacteria to shape host signaling without direct bacterial translocation [14,15]. This concept aligns with a growing consensus that microbiota-derived vesicles are not incidental byproducts but structured mediators of gut homeostasis and disease, with effects that depend strongly on vesicle origin, cargo composition, and the responding host compartment [13,16,17,19,20,56,57]. Accordingly, we contrasted OMVs from the clinically used probiotic *E. coli* Nissle 1917 (EcN) with a commensal comparator (ECOR12) in a paired, core-integrated design spanning cytokines, phenotyping, miRNA profiling, and multivariate integration. In parallel, ECOR63 was included in the extended phenotyping arm for cytokine ELISA and flow cytometry, but was not included in miRNA profiling or integrated PCA/MANOVA due to unavailable matched miRNA measurements. This structure allows mechanistic interpretation to rely on the fully integrated dataset while retaining ECOR63 as a supportive, cytokine-level context.

### 4.1. OMV Heterogeneity Is Measurable and Biologically Meaningful

A necessary premise for interpreting immunological effects is that vesicle preparations reflect biologically plausible OMV populations. Our Cryo-TEM images (Figure 5) supported the expected morphology of OMVs: rounded vesicles with a clearly defined membrane and occasional double-membrane structures consistent with reported OMV architectures and with descriptions of outer–inner membrane vesicles in Gram-negative bacteria [21,22]. The observed nanoscale size range (classically ~20–200 nm, though broader distributions occur across species and preparation conditions) is also compatible with prior work and reinforces that OMVs represent particulate signals capable of traversing mucus and contacting epithelial or immune interfaces [15,21]. Importantly, vesicle-mediated signaling is not restricted to Gram-negative organisms; extracellular vesicles are also produced by Gram-positive bacteria and fungi, emphasizing that vesiculation is a conserved communication strategy [18]. This broader framework strengthens the interpretation that the vesicles observed here plausibly act as cross-kingdom information carriers rather than inert debris [13,74].

Our protein-based quantification approach (Table 1), based on the Lowry assay, provided standardized inputs for downstream comparisons and follows a widely used biochemical method for protein measurement [73]. Nevertheless, protein mass alone cannot capture vesicle number, size distribution, or cargo stoichiometry, and thus should be interpreted as a practical normalization strategy rather than a complete physical characterization. This limitation is widely recognized in OMV/EV studies, where particle-based quantification and orthogonal characterization methods are encouraged to strengthen cross-study comparability [14,15].

Beyond morphology, SDS-PAGE profiles (Figure 6) demonstrated that OMVs from EcN, ECOR12, and ECOR63 contain distinguishable protein patterns, consistent with the concept of selective cargo loading and strain-dependent OMV composition [36,37]. Such differences are expected because OMV biogenesis and protein sorting can depend on outer membrane remodeling, stress responses, and envelope composition, which vary across strains and growth contexts [15]. The proteomic literature further supports that OMV cargo is non-random and often enriched for membrane-associated and immunologically active components, consistent with roles in host interaction and immune modulation [36,37]. However, our comparison also highlights an important interpretive caution: while protein banding patterns can reflect biological differences, they are not a reliable proxy for phylogeny. Although classical *E. coli* phylogenetic grouping (e.g., A, B1, B2, D) provides a valuable framework for interpreting strain background and potential functional tendencies, SDS-PAGE alone cannot resolve phylogeny, particularly when strains share major phylogroups or when vesicle cargo is shaped by environmental regulation rather than lineage alone [34,35]. This reinforces the methodological point that “omics” resolution (proteomics/transcriptomics) and genetic typing are needed to map vesicle composition to strain background with confidence [35,36,37].

### 4.2. OMVs Can Mature Dendritic Cells, but the Direction of the Response Is Strain-Dependent

In the core integrated dataset (iDC control, EcN, ECOR12), OMVs drove DC maturation supported jointly by cytokine release (Figure 7) and maturation-marker shifts by flow cytometry (Figure 8). In the predefined exploratory arm, ECOR63 was included in the extended phenotyping arm to contextualize commensal heterogeneity in cytokine secretion and maturation-marker phenotypes; however, miRNA profiling and cytokine–miRNA multivariate integration were restricted to the core matched dataset (iDC control, EcN, ECOR12) due to unavailable ECOR63 miRNA measurements. This outcome is mechanistically plausible because DCs are designed to integrate microbial signals and translate them into adaptive immune instruction [11,12]. In the gut, DCs operate in continuous dialog with epithelial cells, sampling luminal information directly or indirectly, and shaping T-cell polarization and tolerance programs that maintain homeostasis [6,10]. Disruption of this epithelial–DC crosstalk is therefore a credible route to chronic inflammation and barrier dysfunction [6,10].

From a mechanistic standpoint, OMVs are well-positioned to engage pattern recognition receptors (PRRs) because they can carry pathogen-associated molecular patterns (PAMPs) such as LPS, lipoproteins, and peptidoglycan fragments. PRR activation (e.g., TLRs and NOD-like receptors) initiates innate immune cascades and can also intersect with autophagy-linked immune programs, shaping antigen handling and inflammatory outcomes [5,28]. Consistent with this, prior studies in diverse Gram-negative contexts show that OMVs can modulate epithelial and immune signaling, including NOD1-dependent pathways and DC-mediated T helper polarization [23,24,26]. Importantly, not all OMVs drive the same immunological “tone”: pathogenic OMVs may amplify inflammation and systemic responses, whereas commensal or probiotic OMVs may support balanced defense or immunoregulation depending on cargo and context [16,17,21,22].

Our cytokine profiling (Figure 7) is consistent with this duality. IL-6 and TNF-α are classically associated with inflammatory activation and myeloid signaling cascades, whereas IL-10 is a key immunoregulatory cytokine that helps restrain excessive inflammation and supports tolerance programs in mucosal environments [6,10]. The observation that ECOR12-stimulated DCs exhibited comparatively higher IL-10, while in the extended phenotyping arm, ECOR63 showed higher IL-6 and TNF-α, suggests that commensal strains are not immunologically interchangeable and that OMVs may encode “strain-specific immunological instructions.” This aligns with work showing that vesicles from different *E. coli* backgrounds distinctly modulate human DCs and subsequently shape T-cell responses [55]. It also fits broader models in which microbiota-derived vesicles can act as immunoregulatory agents, potentially contributing to tolerance and homeostasis rather than indiscriminate activation [20,56,57].

Flow cytometry results (Figure 8) provide an orthogonal validation that DCs underwent maturation-associated changes. The increased expression of maturation marker CD83 and the concurrent shifts in monocyte/DC-associated markers are consistent with DC activation in response to microbial stimulation. This is mechanistically coherent with DC biology, where maturation is accompanied by phenotypic reprogramming that supports antigen presentation, costimulatory capacity, and cytokine production [12]. While the observed modulation of CD83/CD14/CD209 together with IL-6/TNF-α versus IL-10 tendencies is consistent with strain-dependent differences in Mo-DC activation states, these data do not establish functional polarization-associated signatures or downstream T-cell instruction. Definitive polarization assignments would require an expanded phenotype/function panel, including additional maturation and trafficking markers (e.g., HLA-DR, CD80, CD86, CCR7), cytokines more directly linked to T-cell skewing (e.g., IL-12p70, IL-23, TGF-β), and functional assays such as antigen presentation capacity, mixed lymphocyte reactions, and Mo-DC–T-cell co-cultures assessing Th1/Th17/Treg polarization. Therefore, we frame the present results as strain-dependent programming of maturation-associated phenotypes and cytokine–miRNA regulatory states, and we propose these functional readouts as the next step to test whether OMV-driven programs translate into distinct adaptive immune outcomes.

### 4.3. EcN OMVs as Postbiotic-like Immunomodulators—And Why “Probiotic” Is Not Equivalent to “Anti-Inflammatory”

EcN is a particularly informative model because it occupies a unique position as a non-pathogenic, clinically used probiotic with documented colonization ability and immunomodulatory potential [29,30]. EcN’s persistence and functional interactions can involve adhesion/colonization factors and fimbriae, which support biofilm formation and intestinal colonization [31]. Additional surface and capsule-associated features can shape epithelial PRR activation and downstream signaling, including MAPK-dependent cytokine induction [32]. This background makes EcN an ideal strain to test the hypothesis that probiotic effects can be mediated not only by live bacteria but also by secreted vesicles as cell-free effectors [14,15].

Consistent with this “postbiotic-like” model, EcN OMVs have been shown to activate mucosal defense programs and to yield anti-inflammatory effects in experimental colitis settings, supporting therapeutic relevance in inflammatory disease contexts [33]. EcN vesicles can also protect epithelial barrier integrity against enteropathogenic insult, reinforcing the concept that vesicles can contribute to barrier resilience [70]. At the same time, EcN is not uniformly anti-inflammatory; rather, its effects can be context-dependent, reflecting the nuanced reality that immune balance often requires controlled activation rather than suppression [29,30]. This nuance also matches broader clinical and nutritional literature on probiotics and IBD, which emphasizes variability across strains, patient contexts, and disease states [7,8]. Our findings that EcN OMVs mature DCs and alter cytokine/miRNA signatures fit well within this framework: EcN may contribute to “trained balance” rather than simple anti-inflammatory silencing, consistent with mucosal immunology principles [6,11].

### 4.4. miRNAs Provide a Mechanistic Layer That Can Reconcile Cytokine Differences with Durable Immune Programming

One of the most important conceptual advances in mucosal immunology is that immune outputs are controlled not only by receptor–ligand interactions and transcription factors, but also by post-transcriptional regulation mediated by microRNAs (miRNAs) [39,40,46]. miRNAs are now understood as essential modulators of DC differentiation and function, shaping the magnitude and duration of innate signaling and cytokine programs [48]. Inflammatory and regulatory circuits can be tuned by miRNA-mediated control of PRR signaling components, cytokine feedback loops, and antigen presentation pathways [44,45]. This is highly relevant for DC biology because DCs are “decision-making” cells that must translate microbial cues into either tolerance-supporting programs or effector responses [11,12].

Our miRNA results (Figure 9 and Figure 10) can be interpreted coherently through this lens. miR-155 is a canonical inflammation-associated miRNA induced during macrophage and DC activation; it modulates cytokine production and has been directly implicated in IL-1 pathway regulation in activated monocyte-derived DCs [41,42]. The observation that miR-155-5p increases with OMV stimulation (EcN and ECOR12) is therefore consistent with OMVs acting as immune-activating signals that engage innate pathways and reprogram DC regulatory circuits [5,28]. Similarly, miR-146 family members are widely described as negative feedback regulators that restrain excessive TLR signaling; IL-10-dependent miR-146b, for example, suppresses TLR4 pathway activity, providing a plausible mechanism by which regulatory cytokine environments stabilize homeostatic immune tone [43,47]. Our finding that miR-146b-5p is induced and may differ between strains (EcN vs. ECOR12) aligns with a model where vesicles can simultaneously trigger activation and encode braking mechanisms—precisely the kind of balanced programming required at mucosal surfaces [6,10].

The let-7 family provides an additional bridge between innate sensing and miRNA regulation. let-7i can regulate TLR4 expression and thereby influence epithelial immune responsiveness to microbial stimuli [52]. Trends toward differential let-7i-3p expression across strains may therefore reflect differences in vesicle cargo that alter PRR pathway setpoints. This possibility is consistent with the broader literature on miRNAs as “fine tuners” of TLR signaling, shaping inflammatory thresholds rather than simply turning pathways on or off [44,45,46].

Crucially, miRNA biology in gut disease extends beyond immune cells: epithelial permeability and barrier integrity are also regulated by miRNAs. miR-29a, in particular, has been associated with increased intestinal permeability in human and experimental contexts, and manipulation of miR-29a can influence tight junction-related proteins such as ZO-1 and claudins [49,50,51]. Thus, our observation that miR-29a-5p is induced and differs between EcN and ECOR12 conditions is biologically meaningful because it suggests that OMVs might influence mucosal outcomes by simultaneously programming DC immunity and barrier-associated regulatory pathways. This integrative view is supported by reviews emphasizing that miRNAs participate in gastrointestinal cell signaling and are implicated in inflammatory bowel disease pathophysiology and immune regulation [58,59].

An especially relevant emerging theme is that bacterial vesicles themselves can carry small RNAs capable of influencing host responses. Small RNAs within OMVs have been proposed as mediators of host–microbe interactions, expanding the mechanistic repertoire beyond proteins and classical PAMPs [53]. Our findings are consistent with this broader hypothesis, particularly when interpreted alongside transcriptomic evidence that gut microbiota secreted vesicles can induce distinct miRNA signatures in DCs [54]. In other words, vesicles may not merely trigger immediate cytokine outputs; they may “write” durable regulatory states into immune cells via miRNA-linked circuits.

### 4.5. Linking Strain-Specific OMV Signaling to Epithelial Interfaces and Disease Relevance

The gut environment is characterized by continuous epithelial exposure to microbial products, requiring robust mechanisms to preserve barrier function while enabling rapid defense. Intestinal epithelial cells regulate barrier architecture, PRR signaling, antimicrobial peptide production, and immune communication, and they therefore represent a key site where OMVs can exert disease-relevant effects [6]. OMV uptake routes such as clathrin-dependent endocytosis have been demonstrated for *E. coli* vesicles in epithelial models, and vesicle internalization can produce divergent outcomes (including DNA damage responses) depending on cargo and context [25]. These mechanisms are not merely technical details: they imply that vesicle effects may be cell-type specific and may differ between epithelial and immune compartments, a point reinforced by broader OMV–host interaction literature [16,17,74].

From a translational perspective, OMVs are therefore plausible candidates for next-generation microbiota-derived interventions (e.g., postbiotics) in gut inflammatory conditions, provided that immunological directionality and safety constraints are established. Clinical and mechanistic reviews emphasize that while probiotics can benefit gastrointestinal disorders, efficacy is strain-specific and context-dependent, and inappropriate immune activation remains a concern [7,8]. This caution becomes especially relevant when one considers that OMVs from some Gram-negative contexts can induce systemic inflammatory phenotypes [21] and that pathogenic vesicles can support virulence and host cell manipulation [22,26]. Importantly, Sat has been reported not to behave as a virulence factor in EcN. Our results support a more precise framing: the question is not whether OMVs are “good” or “bad,” but which vesicle programs (defined by strain, cargo, and recipient cell type) promote balanced mucosal defense versus inflammatory escalation [19,20,57].

Recent work also supports the plausibility of cell-type resolved OMV effects in immune systems beyond DCs. For example, EcN-derived OMVs have been reported to enhance immunomodulation and antimicrobial activity in macrophage contexts [72] and to influence macrophage polarization and colitis outcomes [73]. At epithelial levels, microbiota EVs can modulate nutrient transporters and serotonin related genes via miRNA-associated pathways, indicating that vesicles can re-shape homeostasis relevant gene networks [38]. These data strengthen the interpretation that vesicles can coordinate immune and epithelial axes—precisely the kind of multi-compartment regulation implicated in IBD and related inflammatory conditions [1,2,6].

### 4.6. Why Multivariate Statistics Are Essential for OMV-Immune Datasets

Because OMV responses involve coordinated changes across cytokines, markers, and miRNA panels, multivariate approaches are essential to avoid over-interpreting isolated endpoints. PCA provides a principled way to compress correlated variables into interpretable axes, and biplots allow loadings to be visualized as vectors that clarify which variables drive group separation [60,61,62]. In parallel, clustering and heatmap-based pattern discovery can identify coherent response modules across conditions, supporting mechanistic interpretation rather than single-marker narratives [63,64,65]. Our use of these approaches is therefore aligned with best practices for interpreting complex immunological profiles, particularly when vesicle responses may differ subtly across strains.

Formal multivariate inference also matters. When outcomes are multivariate by nature, MANOVA-based tests provide a framework to evaluate condition effects, and classical work highlights that certain test statistics (e.g., Pillai’s trace) can show favorable robustness properties under assumption deviations [66]. Robust MANOVA alternatives further extend inference reliability when distributions, covariance structures, or outliers challenge classical assumptions [67]. Finally, biological interpretation requires effect-size thinking: statistical significance does not automatically imply biological relevance, and careful reporting of effect sizes and uncertainty is recommended in experimental biology [68]. Even common summary metrics such as partial R^2^ require interpretive caution; partial R^2^ is sensitive to model structure and does not alone validate causality or mechanistic adequacy [69]. Together, these statistical principles justify the analytic strategy used in this study and support more reliable mechanistic claims about strain-specific immunomodulation.

Taken together, our findings support an integrated model in which *E. coli* OMVs act as structured immune stimuli capable of driving Mo-DC maturation and reprogramming miRNA-linked regulatory circuits. Importantly, the integrated cytokine–miRNA “fingerprints” are supported by the core paired dataset (iDC control, EcN OMVs, ECOR12 OMVs; N = 20 donors, N = 60 donor–condition observations), where cytokine outputs, DC phenotyping, miRNA signatures (miR-155, miR-146b, let-7i, miR-29a), and multivariate integration collectively indicate strain-dependent programming rather than uniform activation. To contextualize commensal heterogeneity, ECOR63 OMVs were evaluated in an extended phenotyping arm for cytokine ELISA and flow cytometry, revealing a distinct cytokine/phenotypic tendency relative to EcN and ECOR12; however, miRNA profiling and cytokine–miRNA integrated multivariate analyses were restricted to the core arm due to unavailable matched miRNA measurements for ECOR63. This framework aligns with mechanistic literature on OMV biogenesis and host interaction [15,74], mucosal immunology principles emphasizing epithelial–DC crosstalk [10,11], and evidence that microbiota vesicles can shape cytokine and miRNA landscapes relevant to gut homeostasis and inflammatory disease [20,54,55,56,57]. In the long term, these results reinforce the rationale for moving beyond “microbiota as a black box” toward testable, cell-free vesicle mechanisms that can be quantified, modeled, and potentially engineered for targeted mucosal immunomodulation [5,6,14], while motivating future work to extend matched miRNA profiling and integrated multi-omic analyses to ECOR63 and additional commensal strains.

## 5. Limitations and Future Directions

Several limitations should guide future work. First, while OMV protein normalization is practical [73], particle level quantification (e.g., nanoparticle tracking analysis) and cargo specific assays would strengthen conclusions about dose–response relationships and mechanistic drivers [14,15]. Second, SDS-PAGE provides a coarse view of vesicle composition; proteomics and RNA profiling would better connect cargo differences to immunological outputs [36,37,53]. Third, in vitro DC maturation models capture important biology but cannot fully represent the intestinal microenvironment, where epithelial conditioning, mucus, metabolites, and multi-cellular interactions shape immune thresholds [4,6,10]. Future studies could integrate epithelial–DC co-cultures, macrophage compartments, and barrier readouts to test whether miRNA changes observed here translate into epithelial integrity and tolerance outcomes under inflammatory stress [25,38,72].

Finally, translational relevance requires careful framing within clinical realities of gut disease and exposure routes. IBD and chronic inflammatory disorders have multi-factorial etiologies and can be influenced by infections, diet, and public-health conditions that shape microbial exposures [1,3]. The global food safety perspective underscores that microbial exposures are not purely individualized but occur within population-level systems, an angle that becomes increasingly important when considering microbiota-directed interventions or vesicle-based therapeutics. Thus, OMV-based strategies must be evaluated not only for immunological efficacy but also for safety, reproducibility, and context-dependent effects [16,17,19].

## 6. Conclusions

In conclusion, this study provides mechanistic insight into how outer membrane vesicles (OMVs) released by probiotic and commensal Escherichia coli strains modulate human monocyte-derived dendritic cells (Mo-DCs) across cytokine outputs, maturation phenotypes, and miRNA-linked regulatory circuits. Using a paired donor design (N = 20), we demonstrate in the core integrated dataset (iDC control, EcN OMVs, ECOR12 OMVs; N = 60 donor–condition observations) that OMVs are not inert byproducts but structured stimuli that promote Mo-DC maturation (e.g., increased CD83 with concurrent shifts in CD14/CD209), elicit distinct cytokine profiles (ECOR12 preferentially IL-10–high; EcN higher IL-6/TNF-α tendencies), and induce strain-dependent miRNA responses (miR-155-5p, let-7i-3p, miR-146b-5p, miR-29a-5p). Integrated multivariate analyses (hierarchical clustering and PCA) further support that OMV origin imprints reproducible cytokine–miRNA “fingerprints” rather than a uniform probiotic or commensal response.

To contextualize commensal heterogeneity, ECOR63 OMVs were included in an extended phenotyping arm for cytokine ELISA and flow cytometry (paired within donors; N = 80), revealing a distinct phenotypic/cytokine tendency relative to EcN and ECOR12. However, because matched ECOR63 miRNA measurements were not available, miRNA profiling and cytokine–miRNA integrated multivariate analyses were restricted to the core arm, and conclusions regarding integrated fingerprints apply specifically to EcN versus ECOR12.

Although these patterns indicate strain-dependent Mo-DC maturation and regulatory programming, the study does not directly assess functional DC polarization or T-cell priming; these outcomes should be tested using expanded marker panels and Mo-DC–T-cell co-cultures in future work. Future work should extend the full multi-omic panel to ECOR63 and additional commensal strains, and test functional consequences for T-cell priming, regulatory versus effector polarization, and epithelial barrier outcomes in organoid and in vivo models. Overall, our findings support gut-derived OMVs, including those from probiotic EcN and selected commensals, as tractable non-viable (“postbiotic-like”) effectors whose strain-dependent programs can be quantified to inform rational OMV-based immunomodulation strategies for intestinal inflammatory and immune-mediated disorders.

## Figures and Tables

**Table 1:** Experimental groups and assay coverage for Mo-DC stimulation with OMVs (core integrated arm vs. phenotyping arm).

Assay/Dataset	iDC Control	EcN OMVs	ECOR12 OMVs	ECOR63 OMVs	Notes
OMV characterization (Cryo-TEM, SDS-PAGE)	—	✓	✓	✓	phenotyping arm
Cytokine ELISA (IL-6, TNF-α, IL-10)	✓	✓	✓	✓	phenotyping arm
Flow cytometry (CD14/CD83/CD209)	✓	✓	✓	✓	phenotyping arm
miRNA RT-qPCR (miR-155, let-7i, miR-146b, miR-29a)	✓	✓	✓	—	Core arm only
Integrated multivariate analyses (PCA/heatmap/MANOVA)	✓	✓	✓	—	Core arm only

Note. Inferential statistics treated the donor as the repeated-measures subject (paired design). Mo-DCs were generated from N = 20 independent donors, and each donor contributed paired measurements across conditions. In the core integrated arm (iDC control, EcN OMVs, ECOR12 OMVs), this yielded N = 60 donor–condition observations, which were used for miRNA profiling and cytokine–miRNA integrated multivariate analyses. In the extended phenotyping arm (iDC control, EcN, ECOR12, ECOR63), cytokine ELISA and flow cytometry yielded N = 80 donor–condition observations; ECOR63 was not included in miRNA profiling or integrated multivariate models due to unavailable matched miRNA measurements. Where applicable (ELISA/RT-qPCR), samples were processed in technical triplicate and triplicates were averaged per donor–condition prior to inference.

**Table 2:** Variables evaluated and measurement approaches.

Domain	Variables	Measurement/Output
DC phenotype	CD14, CD83, CD209	Flow cytometry (fluorescent antibody labeling; % positive and/or intensity)
Secreted mediators	IL-6, IL-10, TNF-α	ELISA on culture supernatants (pg/mL)
Post-transcriptional regulation	miRNAs (panel assayed by RT-qPCR; normalized to internal controls/housekeeping)	Relative expression using 2^−ΔΔCt^; log transformed for multivariate analyses when required
OMV input control	Total OMV protein	Lowry protein assay (mg/mL equivalent), used to standardize dosing

Note. The specific antibodies, cytokines, and miRNA RT-qPCR workflow correspond to the study’s established procedures for DC maturation and miRNA validation after OMV stimulation.

**Table 3:** Equipment and acquisition settings used to generate cytokine, miRNA, and phenotyping data.

Data Type	Instrument/Platform	Key Settings/Notes
Flow cytometry	Flow cytometer (FACSCanto™ II; BD Biosciences, San Jose, CA, USA; CCiT-UB cytometry unit)	Antibodies: anti-CD14-FITC, anti-CD83-PE, anti-CD209-APC
Cytokines (ELISA)	ELISA kits (BD Biosciences, San Jose, CA, USA) + microplate spectrophotometry (BMG LABTECH, Ortenberg, Germany)	Readout per manufacturer instructions (typical absorbance at 450 nm)
RNA QC	NanoDrop^®^ ND-1000 (Thermo Fisher Scientific, Wilmington, DE, USA)	A260/A280 ratio used to assess purity
miRNA qPCR	StepOnePlus™ Real-Time PCR System (Applied Biosystems, Foster City, CA, USA)	95 °C 2 min; 40 cycles of 95 °C 10 s and 56 °C 60 s
OMV isolation	Refrigerated centrifuges (Eppendorf SE, Hamburg, Germany) + ultracentrifugation (Beckman Coulter, Brea, CA, USA)	10,000× *g* (30 min, 4 °C); ultracentrifugation RCF = 242,000× *g* (1 h, 4 °C; Type 45 Ti fixed-angle rotor; equivalent to 45,000 rpm)
OMV imaging	Cryo-TEM (FEI, Hillsboro, OR, USA; 200 kV FEG; CCiT-UB microscopy unit)	Cryogenic imaging (~−170 to −175 °C), 200 kV

Note. Instrument access and assay kits correspond to the laboratory workflow and facility resources described in the study documentation.

**Table 4:** Statistical analysis workflow and inference criteria.

Step	Method	Rationale/Reference Support
Data screening	Outlier inspection; distribution checks; transformation (when needed).	Ensures assumptions are evaluated before parametric inference.
Global multivariate test	Donor-blocked MANOVA (response set ~ condition + donor), Pillai trace.	Tests coordinated biomarker shifts while controlling inter-donor variability.
Univariate follow-up	One-way repeated-measures ANOVA (within-subject factor: condition; donor as subject/block) + Holm-adjusted paired post hoc contrasts.	Accounts for the paired donor-derived design and avoids pseudo-replication by treating donors as independent units.
Dimension reduction	PCA + biplot visualization (centered/scaled features).	PCA summarizes correlated immune variables and biplots clarify loadings [60,61,62]. Clustering approaches are widely used to explore structure in multivariate biological datasets.
Pattern discovery	Heatmaps + hierarchical clustering (distance-based); k-means as exploratory option.	Hierarchical clustering and heatmaps reveal response modules [63,64,65]; k-means used as complementary clustering tool.
Reporting	Mean ± SD; effect sizes with CIs when applicable.	Effect-size reporting supports biological interpretation beyond *p*-values [68].

Note. Inferential outcomes (*p*-values, group separations, cluster structures) are reported in the Section 3; Table 4 specifies the a priori decision rules used to evaluate OMV effects.

**Table 5:** Quantification of isolated OMVs by protein measurement.

Strain	Protein Concentration (mg/mL)	Protein Loaded (µg)	Sample Volume for 10 µg (µL)	Total Lane Volume (µL)
EcN	2.898	10	3.45	20
ECOR12	2.744	10	3.64	20
ECOR63	1.518	10	6.58	20

Note. Protein concentration was determined using the Lowry assay [73]. Reported volumes correspond to the preparation of standardized 20 µL mixtures for downstream handling.

**Table 6:** Donor-blocked MANOVA summary (Pillai trace) for integrated biomarkers and biomarker families. Multivariate tests evaluating the effect of strain on (i) the combined cytokine + miRNA set, (ii) cytokines only, and (iii) miRNAs only. Pillai’s trace is reported as a robust multivariate statistic.

Response Set	df (Effect)	Pillai	Approx. F	Num df	Den df	*p*-Value
Cytokines + miRNAs (7 vars)	2	1.9925	1244.85	14	66	3.42 × 10^−74^
Cytokines only (3 vars)	2	1.8868	205.55	6	74	4.75 × 10^−44^
miRNAs only (4 vars)	2	1.9858	1257.69	8	72	4.06 × 10^−74^

**Table 7:** Repeated-measures ANOVAs (donor-blocked) and effect sizes (partial eta-squared, ηp^2^) for the core integrated arm (iDC control, EcN OMVs, ECOR12 OMVs; N = 20 donors). Univariate follow-up tests were performed for each biomarker using the donor as the subject/block.

Biomarker	F (2,38)	*p*-Value	ηp^2^
IL-6	180.21	4.07 × 10^−20^	0.905
IL-10	304.10	4.16 × 10^−24^	0.941
TNF-α	103.62	4.11 × 10^−16^	0.845
miR-155-5p (log2)	1867.48	1.15 × 10^−38^	0.990
let-7i-3p (log2)	1900.41	8.24 × 10^−39^	0.990
miR-146b-5p (log2)	2326.89	1.82 × 10^−40^	0.992
miR-29a-5p (log2)	4205.75	2.55 × 10^−45^	0.996

## Data Availability

The data presented in this study are available on reasonable request from the corresponding author. The data are not publicly available due to privacy/ethical restrictions and the informed-consent terms for human-derived samples.

## Appendix A

**Table A1:** Primers used for miRCURY^®^ LNA^®^ miRNA PCR.

Group 1		Group 2		Group 3		Normalizers	
Name	ID	Name	ID	Name	ID	Name	ID
miR-146b-5p	MIMAT0002809	hsa-miR-29a-5p	MIMAT0004503	hsa-miR-155-5p	MIMAT0000646	hsa−let−7f−5p	MIMAT0000067_1
hsa-miR-24-3p	MIMAT0000080_1	hsa-miR-33a-3p	MIMAT0004506	hsa-miR-146a-5p	MIMAT0000449	hsa−miR−421	MIMAT0003339
hsa-miR-10a-5p	MIMAT0000253	hsa-miR-589-3p	MIMAT0003256	hsa-let-7i-3p	MIMAT0004585		
